# Stochastic Bioimpedance-Based Channel Model of The Human Body for Galvanic Coupling

**DOI:** 10.2478/joeb-2021-0014

**Published:** 2021-12-27

**Authors:** Aaron Roopnarine, Sean A. Rocke

**Affiliations:** 1 Department of Electrical & Computer Engineering, The University of the West Indies, St. Augustine, Trinidad & Tobago

**Keywords:** Human body communications (HBC), bioimpedance, organic communication channels (OCC), propagation modelling

## Abstract

Human body communication (HBC) uses the human body as the channel to transfer data. Extensive work has been done to characterize the human body channel for different HBC techniques and scenarios. However, statistical channel bioimpedance characterisation of human body channels, particularly under dynamic conditions, remains relatively understudied. This paper develops a stochastic fading bioimpedance model for the human body channel using Monte Carlo simulations. Differential body segments were modelled as 2-port networks using ABCD parameters which are functions of bioimpedance based body parameters modelled as random variables. The channel was then modelled as the cascade of these random 2-port networks for different combinations of probability distribution functions (PDFs) assumed for the bioimpedance-based body parameters. The resultant distribution of the cascaded body segments varied for the different assumed bioimpedance based body parameter distributions and differential body segment sizes. However, considering the distribution names that demonstrated a best fit (in the top 3 PDF rankings) with highest frequency under the varying conditions, this paper recommends the distribution names: Generalized Pareto for phase distributions and Log-normal for magnitude distributions for each element in the overall cascaded random variable ABCD matrix.

## Introduction

Human body communication (HBC) uses the human body as the channel to transfer data [1]. Appropriate channel models are extremely important for analysis and optimization of BAN (Body Area Network) performance. The state of the human body (*e.g*., the glucose/other analyte content, position of the electrodes on the body and action being performed by body parts) may be reflected by a correlation with the channel response. This data is invaluable for monitoring patients’ conditions in medical use cases as well as monitoring athletes’ conditions to optimize their performance [2, 3, 4]. The channel response may be used to develop application-specific physical layer technologies, such as Channel Equalization and Automatic Gain Control (AGC) techniques, which aim to improve the communication performance of transceivers used in BANs. Hence, channel models can be leveraged to improve BANs through better error performance, better receiver sensitivity, higher data rate, better channel resilience and power efficiency [5, 6].

Extensive work has been done to characterize HBC channel models for different HBC communication techniques: eHBC (or E-field HBC which involves modulating electric fields through the methods: galvanic coupling and capacitive coupling) and mHBC (or magnetic HBC which involves modulating magneto–quasistatic fields through the method of magnetic induction) [1, 5, 7, 8]. For example, [8] investigated the human body channel for 3 major electrode configurations: on-body, off-body and in-body. There has been notable work as well to develop channel circuit models for the different HBC communication techniques: eHBC (or E-field HBC, which involves modulating electric fields through the methods: galvanic coupling and capacitive coupling) and mHBC (or magnetic HBC which involves modulating magneto-quasistatic fields through the method of magnetic induction) [1, 5, 7, 8]. However, relatively limited emphasis has been placed on the effect statistical properties of the body parameters have on the channel model.

Some have suggested PDFs (Probability Density Functions) for characterizing fading for limited channel variations (sitting, standing and walking) [6, 9] with no single PDF or channel model accounting for dynamic channel state; while others proposed fading margins to account for dynamic channels (*e.g*., [8, 10]). In many instances the investigations focused upon the channel gain, for limited channel variations and electrode positions, with no reference to statistical characterization of fading as a result of these dynamic channel scenarios [11, 10, 12, 13, 14]. Finally, [13, 14] suggested coherence bandwidths based on these limited channel variations.

Consequently, in this paper, stochastic channel modelling for the human body is further investigated using Monte Carlo simulations, with focus on fading for the galvanic coupling HBC technique. It is noted however, that the results equally apply to other HBC techniques as well, due to the common underlying factors which would affect the channel response. The HBC band (*i.e*., 18.375MHz– 23.625MHz) for galvanic communication as HBC is stan-dardised in that region [15]. Consequently, for standardised communication, this model is developed focusing on the HBC band.

## Channel Model

**Figure 1:**
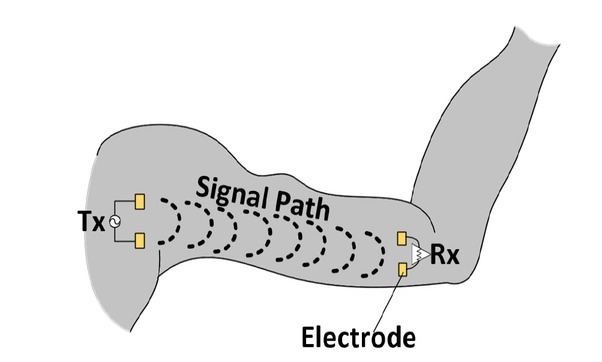
Electrode configuration for galvanic coupling HBC.

Consider the human body channel where currents flow between the electrodes placed on it as shown in Fig. 1, Galvanic coupling involves a differential, modulated signal applied between the transmitter (Tx) electrodes direct in contact with the body. Galvanic currents, are induced in the body and collected by the receiver (Rx) electrodes which are also in contact with the body [5]. The signal path is effected primarily through the skin[1]. Hence, the channel model is dependent upon skin properties.

The literature demonstrates that fading is as a result of channel variations (*e.g*., [8, 11, 10, 12, 13, 14]). Fading is the change in channel gain, and can be related to bioimpedance as this affects the channel response. [1, 16, 17, 18] all show signals sent in the HBC band propagate through the skin. In fact, the electric field, of the propagated signal, in the HBC band, is distributed through different layers of cells; where the skin and fat layers showing the greatest average electric field relative to the other tissue layers [17]. In [1] the authors propose a validated circuit-based static model for the skin for the galvanic coupling HBC technique. Circuit parameters— skin resistance (*R*(*ω*)) and skin admittance (*Y* (*ω*) = *G*(*ω*)+*jB*(*ω*)) which comprises of a shunt circuit between the skin conductance (*G*(*ω*)) and skin susceptance (*B*(*ω*); where *ω* is the angular frequency of the signal propagating through the skin— represent the transfer function of the channel.

Bioimpedance is affected by the body’s biological condition. As investigated in [19], circuit-based parameters depend on electrical properties of the body channel (*i.e*., skin conductivity and permittivity, in particular), which were observed to be roughly frequency flat for the HBC band (18.375MHz–23.625MHz). However, skin circuit parameters also depend on various non-deterministic, time-varying factors such as chemical composition of the body and channel geometry (*e.g*., skin thickness, skin deformation due to movement). Thus, non-deterministic, time-varying factors affect the body channel [20, 21, 22, 23, 24]. For example, [25] showed skin impedance changes when subject to externally-applied forces. In [26] the authors demonstrated skin impedance can vary when skin is damaged through needle punctures. Furthermore, [19] showed skin properties, which affect skin impedance and admittance [1], change when skin is subject to different conditions such as wet or dry scenarios. Bioimpedance is influenced by biological conditions. For example, glucose concentration affects body analyte composition [27]). [28] showed bioimpedance is related to biological state of liver ischemia. [29, 30] showed bioimpdeance body parameters such as relative permittivity differ in the various body parts and subsections such as the dermis and epidermis skin layers.

**Figure 2:**
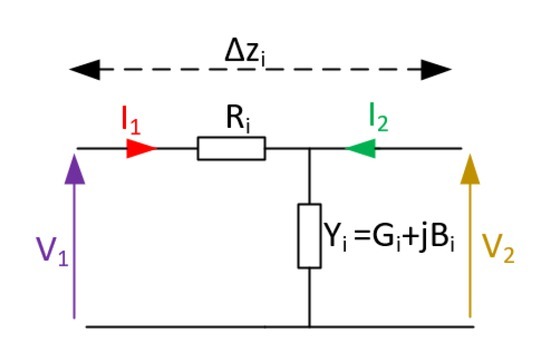
The transmission line model for the *i ^th^* body segment.


Consequently, the human body channel is taken as a non-deterministic, time-varying channel. In this work, the human body is modelled as a lossy transmission line subdivided into sections, Δ*z_i_* (*i.e*., the *i ^th^* body segment), as seen in Fig. 2, Thus, each differential/discretized section, of length Δ*z_i_* , can be modelled as an ABCD 2-port network given by:

(1) $$\begin{array}{rl} \Gamma_{i} & =\left[\begin{array}{cc} A_{i} & B_{i}\\ C_{i} & D_{i} \end{array}\right]=\left[\begin{array}{cc} 1+\frac{R_{i}}{Y_{i}} & R_{i}\\ \frac{1}{Y_{i}} & 1 \end{array}\right]\\ & =\left[\begin{array}{cc} 1+\frac{R_{i}}{G_{i}+jB_{i}} & R_{i}\\ \frac{1}{G_{i}+jB_{i}} & 1 \end{array}\right]. \end{array}$$

This follows from [1], where the *i ^th^* body differential segment parameters in (1) are:

- *Y_i_* = *G_i_* + *jB_i_* , the admittance of the channel composed of the tissue conductance (*G_i_* ) and susceptance (*B_i_* ), which account for coupling between the conductive pathways of the tissue; and
- *R_i_* , the shunt resistance of the tissue which accounts for signal propagation between cells.

Since the skin and body are inhomogeneous [19], this model simplifies the incorporation of this property by dividing the skin into homogeneous, differential sections; each with non-deterministic time-varying parameters.

This segmented transmission line model form allows modelling of random variations due to causes of fading (*e.g*., movement and biological conditions in particular). Hence, the on-body human body channel can be represented as a random process matrix, ⌈*i* , with probability density functions (PDFs) for the parameters that each of its elements depend on – *R_i_* , *G_i_* and *B_i_* (denoted by *f_Ri_* , *f_Gi_* and *f_Bi_* respectively). Each Δ*z_i_* is initially, assumed to be independent and identically distributed (IID). Hence, the PDFs for each *R_i_* , *G_i_* and *B_i_* become *R*, *G* and *B* respectively with PDFs: *f_R_*, *f_G_* and *f_B_* respectively. To the best of the authors’ knowledge, based on the extent of the literature surveyed, these PDFs have not been characterized.

Thus, ⌈*i* can be considered as a continuous–valued, discrete–parameter random matrix, containing corresponding random processes: *A_i_* , *B_i_* , *C_i_* and *D_i_* , with index set *i* = *{*1*, . . . ,N}*, where *N* is the number of Δ*z_i_* segments that constitute the channel. Hence, the channel length, $I=\sum_{i=1}^{N}\Delta z_{i}$where Δ*z_i_* is the length of the *i ^th^* segment. Since each Δ*z_i_* segment is assumed to be IID, then this process can be considered stationary as well [31]. Furthermore, this implies that one realization can be used to characterize at least the mean and auto-correlation, since it satisfies the index invariance (in this case, space invariance for short time intervals) condition, which points to ⌈*_i_* being ergodic in the mean and auto-correlation [31].

Given the use of ABCD network modelling, the entire human body channel can be considered as an aggregate 2-port network equivalent to the cascade of *N* differential 2-port networks, to give the overall HBC network representation under matrix multiplication as follows:


(2) $$\Gamma =\prod_{i=1}^{N}\Gamma_{i}$$

This research aims to statistically characterise ⌈ through numerical simulations based on circuit-based electrical body parameters.

## Methodology

Even under the simplest assumptions for the channel segments, statistical models for the overall ABCD matrix for the channel may not be analytically tractable. Recall work has yet been done investigating the effect of random variations in biological state on the channel response. The authors’ approach involves a Monte Carlo simulation-based investigation of various candidate models based upon assumptions for the PDFs for the channel segments which make sense practically. Distributions were assumed for *f_R_*, *f_G_* and *f_B_* with parameters determined through the literature surveyed. Segments were assumed to be independent and identically distributed. It is noted, however, that correlation can be factored in.

Through (1) and (2), the cascaded ABCD parameters of the human body channel may tend towards a distribution based on the assumed PDFs. The following PDFs for these parameters are considered:

- *Normal Distribution*. This was chosen as a candidate for segments based upon scenarios where there may be a central tendency with a spread that can be approximated by the Gaussian distribution. Thus: $R∼N\left(\mu_{R},\sigma_{R}^{2}\right),G∼N\left(\mu_{G},\sigma_{G}^{2}\right)\text{ and }B∼$$N\left(\mu_{B},\sigma_{B}^{2}\right)$where each PDF contain finite means $\left(\mu_{R},\mu_{G}\text{ and }\mu_{B}\right)$and variances $\left(\sigma_{R}^{2},\sigma_{G}^{2}\text{ and }\sigma_{B}^{2}\right).$The mean values, for *R*, *G* and *B*, were selected using the formulae for these parameters in [1]; with the values for the skin’s permittivity (*ε*(*ω*)) and conductivity (*σ*(*ω*)) taken from the study done by [30] for the HBC band. The variances were set to different multiples (*×*1, *×*2 and *×*3 times) of the means to account for parameter changes seen in the literature surveyed (*e.g*., [20, 21, 22, 23, 24] which showed skin resistance changes for different scenarios).
- *Uniform Distribution*. This was chosen as a candidate for segments based upon scenarios where the range of values for *R*, *G* and *B* may be equally likely. Thus: *R ∼ U*(*R_min_,R_max_* ), *G ∼ U*(*G_min_,G_max_* ) and *B ∼ U*(*B_min_,B_max_* ), where the minimum (*R_min_*, *G_min_* and *B_min_*) and maximum (*R_max_* , *G_max_* and *B_max_* ) values for each PDF are also finite. The minimum values were selected as 0 for each parameter as these are possible based on the literature surveyed. The maximums were set to different multiples of the means determined from the Normal distribution case as done for the variances.
- *Gamma Distribution*. Since *f_R_*, *f_G_* and *f_B_* may take on different shapes, the Gamma distribution was considered. This distribution is typically used to
- model wait times between Poisson distributed events; i.e. it models sums of exponentially distributed random variables. Thus, it can be linked to this use case. Furthermore, this use case takes advantage of this distribution’s versatility in shape through manipulation of the shape parameters: *a* and *b*, Thus: *R ∼ Gamma*(*a_R_, b_R_*), *G ∼ Gamma*(*a_G_, b_G_*) and *B ∼ Gamma*(*a_B_, b_B_*), where the shape parameters are finite. The means and variances derived for the Normal distribution were used to derive the shape parameters of this distribution where: *mean* = *ab* and *variance* = *ab*^2^.

Note, these PDFs were chosen as they are commonly used for phenomena that have the traits stated above. However, the approach adopted can be generalized to accommodate other distributions as well (*e.g*., Rayleigh, Log-normal).

Now consider each differential section of skin to be of dimensions: *t ×*Δ*z_L_ ×*Δ*z_L_*, where *t* is skin thickness and Δ*z_L_* is the length of a differential segment (Δ*z_i_* ). *R*, *G* and *B* are determined based upon [1]. Since each *R*, *G* and *B* are random variables, and the size Δ*z_L_* does not affect the values of *R*, *G* and *B* [1], the same PDFs for these parameters can be assumed for all values of Δ*z_L_*, Table 1 shows the PDFs assumed for *R*, *G* and *B* whose parameters were estimated based on the literature surveyed.

**Table 1:** The PDFs assumed for *R*, *G* and *B*.

PDF Test Case	R	G	B
A	*N*(2.38*E* + 03, 2.38*E* + 03)	*N*(4.20*E −* 04, 2.40*e −* 04)	*N*(2.85*E* + 07, 2.85*E* + 07)
B	*N*(2.38*E* + 03, 4.76*E* + 03)	*N*(4.20*E −* 04, 8.40*E −* 04)	*N*(2.85*E* + 07, 5.70*E* + 07)
C	*N*(2.38*E* + 03, 7.14*E* + 03)	*N*(4.20*E −* 04, 1.26*E −* 03)	*N*(2.85*E* + 07, 8.55*E* + 07)
D	*U*(0, 2.38*E* + 03)	*U*(0, 2.40*e −* 04)	*U*(0, 2.85*E* + 07)
E	*U*(0, 4.76*E* + 03)	*U*(0, 8.40*E −* 04)	*U*(0, 5.70*E* + 07)
F	*U*(0, 7.14*E* + 03)	*U*(0, 1.26*E −* 03)	*U*(0, 8.55*E* + 07)
G	*Gamma*(2.3810*E* + 03, 1)	*Gamma*(4.2000*E −* 04, 1)	*Gamma*(2.8501*E* + 07, 1)
H	*Gamma*(1.1905*E* + 03, 2)	*Gamma*(2.1000*E −* 04, 2)	*Gamma*(1.4250*E* + 07, 2)
I	*Gamma*(7.9365*E* + 02, 3)	*Gamma*(1.4000*E −* 04, 3)	*Gamma*(9.5002*E* + 06, 3)

**Figure 3:**
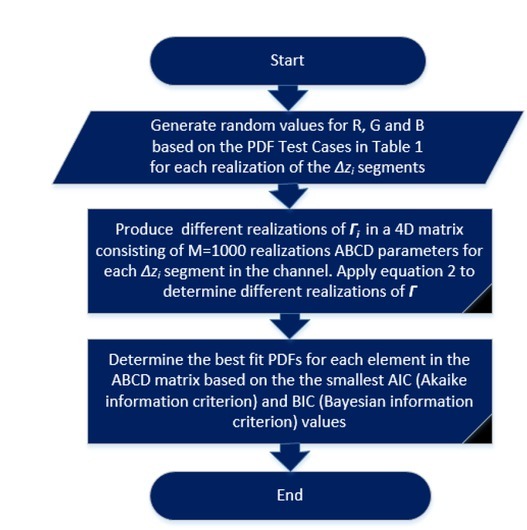
Algorithm implemented in MATLAB to determine the resultant distributions for ⌈ for the different combinations of PDFs for *R*, *G* and *B*.

Fig. 3 shows the algorithm followed to plot the PDFs of the elements of the resultant cascade, ⌈, using the different combinations of the PDFs for *R*, *G* and *B* in Table1 for different Δ*z_L_* lengths (0.01mm to 10mm). These PDFs can be used to represent the dynamic channel.

## Results and Analysis

This section presents the findings from evaluation of the stochastic model through simulation and empirical analysis.

### Simulation of Stochastic Model

**Figure 4:**
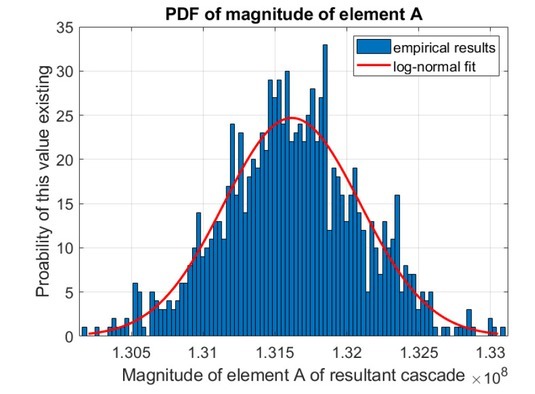
Sample PDF derived for the magnitude of element A of the resultant cascade, ⌈, for test case A with Δ*z_L_* = 0.1*mm*.

A repository of the results obtained from the simulation process can be accessed online [32]. Fig. 4 shows an example PDF plot approximation for the magnitude of element A of the resultant cascade, ⌈, for test case A with Δ*z_L_* = 0.1*mm*.

**Figure 5:**
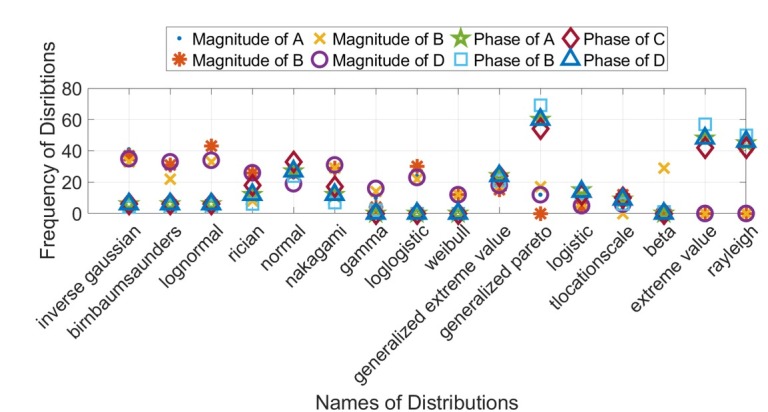
Frequency of Distribution Names (Considering the Top 3 Best Fit Distributions) for the Cascaded ABCD Random Variable Matrix Elements Considering both Magnitude and Phase.

No single distribution was found to be the best fit for all variations of the inputted parameters. Table 2 and Fig. 5 show the frequency of distribution names (considering the top 3 best-fit distributions) for ⌈’s elements considering both magnitude and phase. Distribution names can be assumed for the magnitude and phase of each element of ⌈ based on the distribution names that occur with the highest frequency under the different PDF test cases and Δ*z_L_* sizes. Thus, based on this criteria, the following distribution names can be assumed for the magnitude and phase of each element of the cascaded random variable ABCD matrix, ⌈.

**Table 2:** The percentage of times each distribution fell within the top 3 best-fit candidates for each element of the cascaded random variable ABCD matrix, ⌈, BS-Birnbaum-Saunders. GEV-Generalized Extreme Value, GP-Generalized Pareto.

Distribution Name	A		B		C		D	
	Magnitude	Phase	Magnitude	Phase	Magnitude	Phase	Magnitude	Phase
Inverse Gaussian	15.19	2.22	13.7	1.48	12.22	2.22	12.96	2.22
BS	11.85	2.22	11.48	1.85	8.15	2.22	12.22	2.22
Lognormal	15.19	2.22	15.93	1.85	12.22	2.22	12.59	2.22
Rician	8.89	4.44	9.63	2.22	2.96	6.67	9.63	4.44
Normal	8.52	10	10	8.89	9.63	12.22	7.04	10
Nakagami	10	4.44	10.74	2.59	10.74	6.3	11.48	4.44
Gamma	3.7	0	1.48	0.74	5.19	0	5.93	0
Log-logistic	8.89	0	11.11	0	8.15	0	8.52	0
Weibull	4.44	0	4.44	0	4.44	0	4.44	0
GEV	6.67	8.89	5.56	7.78	6.67	8.89	6.67	8.89
GP	4.44	22.22	0	25.56	6.3	20	4.44	22.22
Logistic	1.11	5.56	1.48	4.81	2.59	4.44	1.85	5.19
T Location-Scale	1.11	3.33	4.44	2.22	0	3.7	2.22	3.33
Beta	0	0	0	0.37	10.74	0	0	0
Extreme Value	0	17.78	0	21.11	0	15.56	0	17.78
Rayleigh	0	16.67	0	18.52	0	15.56	0	17.04

- Magnitude of Elements: *Log-normal*, Inverse Gaussian demonstrated a marginally better fit than with the Log-normal distribution only in the case of the element D. However, this is small enough to validate the Log-normal best fit for element D.
- Phase of Elements: *Generalized Pareto*.

NOTE: Only distribution names are suggested since in cases where they rank in the top 3 best fit PDFs, their shape parameters vary under the different conditions investigated.

Log-normal distributions are used in cases where a large number of individual effects, not strictly independent, act together on a signal [33]. Multiple factors, outlined in section , can affect the channel. Consequently, the Log-normal distribution demonstrated the best-fit as according to the AIC and BIC criteria for the magnitude of each element in ⌈, Since the channel loss is element A in decibels, the channel loss can be classified as possessing a normal fading characteristic validating the Central Limit Theorem (CLT).

The mean of the channel losses for the distributions (for the magnitude of element A where Δ*z_L_* = 0.1*mm* in test cases A-C) fall within measured static channel loss values in [1] and [12] under similar conditions simulated. Thus, to some extent, the PDFs derived for the magnitude of element A where Δ*z_L_* = 0.1*mm* in test cases A-C are validated. This gives credence to the channel length: Δ*z_L_* = 0.1*mm* being the best candidate to be used in the stochastic channel model of the human body for galvanic coupling.

Some additional findings from the simulated results are:

- Increasing Δ*z_L_* decreases the mean of the distributions for the magnitude of the elements of the cascaded random variable ABCD matrix, ⌈, This is intuitive since a higher Δ*z_L_* length corresponds to a smaller cascade length and hence a smaller summation term for the cascaded element.
- Increasing Δ*z_L_* induces slight changes in the distribution parameters for the phases of the elements of ⌈.
- Increasing channel length changes the distributions by increasing their mean. Since the distribution of the parameters for *R*, *G* and *B* are the same for all Δ*z_i_* segments for different Δ*z_L_* sizes, increasing the channel length will be equivalent to increasing the number of Δ*z_i_* samples. In other words, the results obtained for a Δ*z_L_* of 1mm will be the equivalent to increasing the channel for a Δ*z_L_* case of 10mm by a factor of 10.
- The magnitude and phase of each element of ⌈ are dependent. This was determined through finding the Pearson correlation coefficient between the magnitude and phase. This is a non zero value for all PDF test cases— of *R*, *G* and *B*— and Δ*z_L_* sizes except for 3 cases: for cases D,E and F for element C where Δ*z_L_* = 0.05*m*, Thus, magnitude and phase are correlated for each element in the cascaded random variable ABCD matrix except for 3 cases: for cases D,E and F for element C where Δ*z_L_* = 0.05*m*, A counter example was found for each of these uncorrelated cases that showed no independence; i.e. the joint PDF of magnitude and phase did not equate to the product of the marginal PDFs of magnitude and phase. Hence, the magnitude and phase are dependent for all elements in ⌈ under all conditions. Consequently, there is no further need to investigate independence between magnitude and phase.
- For all test cases, except test cases D-F (which considers uniform distributions for *R*, *G* and *B*), the magnitude and phase demonstrate high negative correlation. For cases D-F there is relatively low correlation between the magnitude and phase. The magnitude and phase became more negatively correlated as the Δ*z_L_* size increases for cases D-F. Thus, the magnitude and phase of each element become less correlated as Δ*z_L_* increased only for cases where there are uniform distributions for *R*, *G* and *B*, Since the distribution of the parameters for *R*, *G* and *B* are the same for the Δ*z_i_* segments for different Δ*z_L_* sizes, increasing the channel length will be equivalent to increasing the number of Δ*z_i_* samples. In other words, the results obtained for a Δ*z_L_* of 1mm will be the equivalent to increasing the channel for a Δ*z_L_* case of 10mm by a factor of 10, Thus, as the channel length increases, the magnitude and phase become more correlated for each of the elements in ⌈ if uniform distributions are assumed for *R*, *G* and *B*.
- Distributions typically used for fitting tail ends of other distributions—extreme value and gereralized Pareto — fit better for the phase elements when compared to the other distribution fits. The Rayleigh distribution fit relatively well distributions associated with the phase elements for cases where Δ*z_L_* = 0.01*mm*, Rayleigh distributions are used to model complex Gaussian random events making it a suitable candidate for modelling the phase elements in ⌈ [33]. These 3 distributions have similar shapes which points to an empirically derived phase element distribution resembling that typically used to measure tail-ended distributions.

## Conclusion

Stochastic models of the channel were produced for different channel model configurations via a simulation–based approach (Δ*z_L_* size and input PDF combination cases that describe ⌈*_i_* outlined in Table 1). These stochastic models changed as the channel model configurations changed suggesting that there was no single universal approximate distribution fading model. Further, the investigations focussed upon scenarios without channel segment correlation. Considering the distribution names that demonstrated a best fit (in the top 3 PDF rankings) with highest frequency under the different channel model configurations, this paper recommends the following distributions for consideration when modelling channel fading for dynamic HBC scenarios: Generalized Pareto for phase distributions and Log-normal for magnitude distributions for each element in the cascaded random variable ABCD matrix. Additionally, there is a need to investigate these findings empirically. Furthermore, the method for determining the stochastic fading model for the HBC technique, galvanic coupling, in this paper can be adopted in future work for other communication techniques (*i.e*., capacitive coupling or magnetic induction).
